# Association of dietary quality and mortality in the non-alcoholic fatty liver disease and advanced fibrosis populations: NHANES 2005–2018

**DOI:** 10.3389/fnut.2025.1507342

**Published:** 2025-01-23

**Authors:** Xingyong Huang, Xiaoyue Zhang, Xuanyu Hao, Tingting Wang, Peng Wu, Lufan Shen, Yuanyuan Yang, Wenyu Wan, Kai Zhang

**Affiliations:** ^1^Department of Gastroenterology, Endoscopic Center, Shengjing Hospital of China Medical University, Shenyang, China; ^2^Key Laboratory of Immunodermatology, Ministry of Education, Department of Dermatology, The First Hospital of China Medical University, Shenyang, China; ^3^Key Laboratory of Immunodermatology, National Health Commission of the People's Republic of China, The First Hospital of China Medical University, Shenyang, China; ^4^National and Local Joint Engineering Research Center of Immunodermatological Theranostics, The First Hospital of China Medical University, Shenyang, China

**Keywords:** dietary quality indexes, mortality, NAFLD, advanced fibrosis, NHANES

## Abstract

**Background:**

Nonalcoholic fatty liver disease (NAFLD) has emerged as a significant global health concern, with advanced fibrosis increasing mortality risks. Despite the abundance of dietary guidelines for managing NAFLD, the precise impact of diet quality on mortality among individuals with advanced fibrosis remains elusive. This study aims to explore the influence of five dietary quality indexes on mortality among NAFLD patients and advanced fibrosis patients.

**Methods:**

This study utilized data from the National Health and Nutrition Examination Survey (NHANES) spanning from 2005 to 2018 to assess dietary quality based on the Alternate Mediterranean Diet (aMED), Healthy Eating Index-2020 (HEI-2020), Dietary Approach to Stop Hypertension (DASH), Alternate Healthy Eating Index (AHEI), and Dietary Inflammatory Index (DII). Weighted Cox proportional hazard regression models along with restricted cubic splines and subgroup analyses were employed in this study.

**Results:**

The analysis encompassed 3,634 NAFLD patients. After a median follow-up of 89 months, it was found that higher scores on the aMED (HR 0.814, 95% CI 0.681–0.972), HEI-2020 (HR 0.984, 95% CI 0.972–0.997), DASH (HR 0.930, 95% CI 0.883–0.979), and AHEI (HR 0.980, 95% CI 0.966–0.995) were associated with lower mortality risks, while DII scores (HR 1.280, 95% CI 1.098–1.493) indicated an increased risk of mortality. Additionally, a nonlinear relationship was identified solely between AHEI scores and all-cause mortality in NAFLD patients. Notably, among patients with advanced fibrosis, HEI-2020 as a categorical variable (T3: HR 0.519, 95% CI 0.280–0.964), DASH as a continuous variable (continuous: HR 0.921, 95% CI 0.849–0.999), AHEI (continuous: HR 0.971, 95% CI 0.945–0.997; T2: HR 0.545, 95% CI 0.310–0.960; T3: HR 0.444, 95% CI 0.245–0.804), and DII (continuous: HR 1.311, 95% CI 1.121–1.534; T3: HR 2.772, 95% CI 1.477–5.202) exhibited significant associations with all-cause mortality. Subgroup analyses revealed an interaction between AHEI scores and sex among NAFLD patients, where higher AHEI scores correlated with lower all-cause mortality in females, but no such association was observed in males. For other dietary quality, subgroup analyses indicated that their relationships with mortality were robust.

**Conclusion:**

Our study suggests that a high-quality diet could potentially mitigate mortality risk in both NAFLD and advanced fibrosis patients.

## 1 Introduction

Non-alcoholic fatty liver disease (NAFLD) stands out as one of the most common contributors to chronic hepatopathy globally, affecting around a quarter of the world’s population (1). Epidemiological evidence reveals that the prevalence of NAFLD ranges from 14 to 32% across various regions worldwide, with the highest rates observed in South America and the Middle East and the lowest prevalence found in Africa (2). NAFLD commences with the accumulation of fat in the liver. In clinical practice, it is generally characterized by the presence of ≥5% hepatic fat accumulation in the absence of excessive alcohol consumption and other known causes of fatty liver, such as viral infections, medications, and autoimmune conditions. The mortality associated with NAFLD is closely tied to complications such as cirrhosis, cancer, type 2 diabetes, and cardiovascular diseases (3).

NAFLD encompasses a broad spectrum, including nonalcoholic fatty liver, nonalcoholic steatohepatitis (NASH), and cirrhosis (4). Certain NAFLD individuals exhibit advanced fibrosis, staged according to liver biopsy outcomes, where those with stage 3 or 4 liver fibrosis are diagnosed with advanced fibrosis (5, 6). Many patients with NAFLD or NASH present asymptomatically. Elevated alanine aminotransferase (ALT) and aspartate aminotransferase (AST) levels are common findings in these patients; however, these enzyme levels show little correlation with disease severity. ALT and AST serve as markers of hepatocellular injury, with ALT being more specific to liver damage, while AST can be influenced by extrinsic factors such as muscle injury. Notably, an increased AST-to-ALT ratio is closely associated with advanced liver fibrosis and is incorporated into several composite risk scores for fibrosis assessment (7). Research indicates that advanced fibrosis serves as a robust prognostic indicator in NAFLD patients. Compared to individuals with stage 0–2 fibrosis, those with advanced fibrosis demonstrate significantly shorter survival times and an elevated risk of overall mortality (5, 8). NAFLD and advanced fibrosis pose a substantial global health burden and are acknowledged as the fastest-growing causes of liver-related mortality worldwide (9). Moreover, model projections suggest that the economic burden of these conditions on healthcare systems may escalate exponentially in the coming years (10). Given this critical situation, there is an urgent necessity to deepen our understanding of the factors contributing to the increasing incidence of NAFLD and its adverse outcomes. Strengthening public health prevention strategies and improving patient education and management are critical steps in mitigating, or even reversing, the escalating tide of these diseases.

The development of NAFLD is currently believed to be the result of complex interactions between environmental factors and genetic risk factors. Specifically, poor dietary habits, sedentary lifestyles, and socioeconomic factors are tightly linked to the occurrence and advancement of NAFLD (11, 12). Due to the absence of approved effective pharmacological treatments for NAFLD, in recent years, several studies have incorporated recommendations for lifestyle changes and dietary modifications in the management of NAFLD (13, 14). Evidence suggests that high-quality diets are associated with a reduced risk of NAFLD. However, the relationship between diet quality and mortality in NAFLD patients, especially those with advanced fibrosis, remains limited and inconsistent, necessitating further investigation (15, 16). Consequently, further exploration is imperative to investigate the connection between diet quality and mortality rates among NAFLD patients, especially those with advanced fibrosis.

Currently, an increasing number of studies are adopting a comprehensive approach to dietary patterns, recognizing the synergistic effects of food consumption on health outcomes rather than focusing solely on single foods or nutrients (17, 18). In our research, we similarly employed this strategy, utilizing various indexes to assess the overall dietary quality of participants, including the Mediterranean diet (MED) score, the Healthy Eating Index-2020 (HEI-2020), the Alternative Healthy Eating Index (AHEI), the Dietary Approaches to Stop Hypertension (DASH) diet, and the Dietary Inflammatory Index (DII) (19–22). Consequently, this observational study aimed to examine the potential impact of different dietary quality indexes on the mortality of NAFLD patients and further explore the influence of diet quality on the mortality of those with advanced fibrosis.

## 2 Methods

### 2.1 Study population

The population data for this research were sourced from the NHANES dataset covering 2005–2018. NHANES is a cross-sectional survey that serves a crucial role in disease prevention and enhancing the nation’s overall health by collecting comprehensive health and nutritional data across the US. For more detailed information about NHANES, please refer to their official website (23).

**Figure 1:**
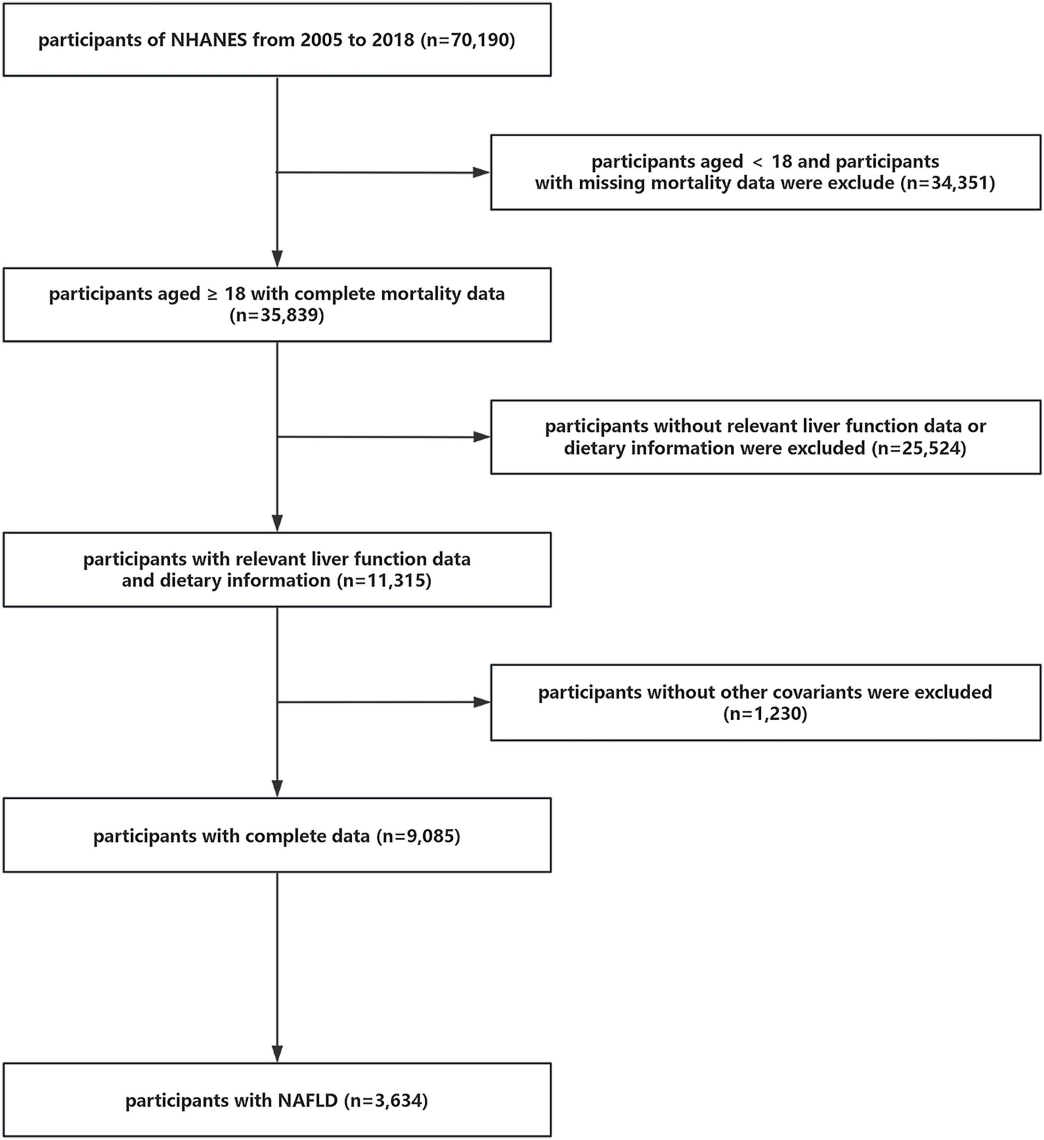
Flow chart for participant inclusion and exclusion.

A total of 70,190 individuals initially participated in the study. Firstly, participants <18 years of age and without mortality data were eliminated (*n* = 34,351). Subsequently, individuals who lacked dietary information or data to determine whether they had NAFLD or advanced fibrosis were also ruled out (*n* = 25,524). Afterwards, we excluded participants without covariate data (*n* = 1,230). Ultimately, 3,634 individuals with NAFLD were selected from among the remaining participants for this study. The detailed process of inclusion and exclusion is illustrated in Figure 1.

### 2.2 Definition of NAFLD and advanced fibrosis

In this study, the fatty liver index (FLI) was primarily applied to determine whether participants had NAFLD. FLI is a score that combines triglycerides (TG), body mass index (BMI), gamma-glutamyltransferase (GGT), and other parameters to evaluate the degree of hepatic steatosis in participants, and it can accurately identify NAFLD (24, 25). The formula for calculating FLI was as follows:


$$FLI=\frac{e^{0.953\times \ln\left(TG\right)+0.139\times BMI+0.718\times \ln\left(GGT\right)+0.05\times \mathrm{waist circumference}-15.745}}{1+e^{0.953\times \ln\left(TG\right)+0.139\times BMI+0.718\times \ln\left(GGT\right)+0.05\times \mathrm{waist circumference}-15.745}}\times 100$$

If a participant had an FLI ≥ 60, did not have hepatitis B or hepatitis C, and did not consume excessive alcohol (defined as alcohol consumption ≥30 g/day for men and ≥ 20 g/day for women (26, 27)), then he or she was considered to have NAFLD.

Whether a participant had advanced fibrosis was determined based on three serological composite scores: the NAFLD fibrosis score (NFS), Fibrosis 4 Score (FIB-4), and AST to platelet ratio (APRI). These scores were created to assess the severity of liver fibrosis, and their reliability has been well established (7, 28–30). A participant was defined as having advanced fibrosis if the APRI was >1, or the FIB-4 was >2.67, or the NFS was >0.676 (7). The calculation formulas for these three indexes were as follows:


$$\begin{array}{l} NFS=-1.675+0.037\times \mathrm{age}+0.094\times BMI+1.13\\ \times \mathrm{impaired fasting glucose}\;\left(IFG\right)+0.99\times \frac{AST}{\mathrm{ALT}}\\ -0.013\times \mathrm{platelet counts}-0.66\times \mathrm{albumin} \end{array}$$

$$\mathrm{FIB}-4=\frac{\mathrm{age}\times AST}{\mathrm{platelet counts}\times \sqrt{\mathrm{ALT}}}$$

$$\mathrm{APRI}=\frac{AST÷\mathrm{upper limit of normal}\times 100}{\mathrm{platelet counts}}$$

AST = 40 U/L was used as the upper limit of normal (31, 32).

### 2.3 Mortality data

The study participants were followed up until their deaths or the conclusion of the follow-up period on December 31, 2019. Their mortality information was collected by linking to the National Death Index, which was available for download on the website of the National Center for Health Statistics (33). All-cause mortality encompassed deaths from various causes, categorized into cardiovascular diseases, malignant neoplasms, chronic lower respiratory conditions, diabetes mellitus, and others. In this study, we analyzed all-cause mortality and cardiovascular mortality among participants.

### 2.4 Dietary quality indexes

The information used to calculate the dietary quality indexes in this study was obtained from dietary data in NHANES. All NHANES participants underwent two 24-h dietary recall interviews conducted by professional interviewers, one at the mobile examination center (MEC) and the second through a follow-up phone interview conducted 3 to 10 days later. Both dietary recall interviews’ data were analyzed to calculate the participants’ dietary quality indexes.

We utilized the alternate Mediterranean Diet (aMED) score to evaluate the adherence to the MED. The aMED score was calculated based on the intake of the nine dietary components. Individuals were allocated 1 point for each component if their intake exceeded the median, apart from red and processed meat and alcohol. For red and processed meat, 1 point was assigned if the intake was below the median, and for alcohol, 1 point was given if consumed moderately. The total score varied from 0 to 9, with a higher score reflecting better adherence to the MED diet (34–36). The HEI-2020 score was based on 13 components, including adequacy components and moderation components. Participants received higher scores for higher intakes of these adequacy components, while for the moderation components, participants got higher scores for intakes within a certain range. The score ran from 0 to 100, where elevated scores denoted better dietary quality (37, 38). The DASH score contained eight components and participants were ranked according to the intake levels of these components, divided into quintiles. For whole grains, fruits, vegetables, nuts and legumes, and low-fat dairy products, participants in the 1st quintile got 1 point, while those in the 5th quintile acquired 5 points. Conversely, for the remaining components, participants in the 1st quintile received 5 points, while those in the 5th quintile received 1 point. The total DASH score ranged from 8 to 40, with augmented scores corresponding to enhanced compliance to the DASH diet (39, 40). The AHEI score varied from 0 to 110, comprising 11 elements, each scored on a scale of 0 to 10. Higher scores indicated proper intake levels of these elements, reflecting better diet quality (41). The DII score utilized 45 food parameters to evaluate the dietary inflammatory potential of participants. It showed the participant’s dietary inflammatory level based on its association with inflammatory biomarkers such as Interleukin-6 and Interleukin-10. Since the dietary data from NHANES did not include all 45 food parameters, the DII scores in this study were calculated from the 28 nutrients or food components available in the NHANES database (22, 42, 43).

We utilized the R package “dietaryindex” to help in the calculation of these dietary quality indexes (44).

### 2.5 Covariates

Aiming to ensure the rigor and scientific validity of the research, the following covariates were included: age, sex (male/female), race (non-Hispanic White/non-Hispanic Black/Mexican American/other Hispanic/other races), BMI, hypertension condition, and diabetes condition.

Hypertension was judged by self-report and systolic and diastolic blood pressure measured at the MEC. We defined those who self-reported as “yes” or had systolic blood pressure ≥ 140 mmHg or diastolic blood pressure ≥ 90 mmHg as hypertensive patients (45). Similarly, if participants reported having diabetes or taking antidiabetic drugs, if their glycated hemoglobin (GHB) was ≥6.5%, or if their fasting plasma glucose level was ≥126 mg/dL, we considered them to be diabetic patients (46).

### 2.6 Statistical methods

To guarantee the precision of our study, we employed the data weighting methods recommended by NHANES to address the complex and multistage sampling design of the NHANES data.

For baseline characteristics in all patients with NAFLD, continuous variables were exhibited as means and standard errors (SEs), while categorical variables were represented as non-weighted numbers and weighted percentages. In addition, BMI and the five dietary quality indexes were analyzed using both continuous and categorical variables. BMI was divided into three groups: <25 kg/m^2^, 25 to <30 kg/m^2^, and ≥ 30 kg/m^2^. Each dietary quality index was categorized into three groups from low to high according to tertiles: T1, T2, and T3. We also presented the baseline characteristics according to the tertiles of five dietary quality indexes. We utilized correlation analysis to examine the associations among these five dietary quality indexes and applied multivariate linear regression models to explore the associations between these dietary quality indexes and ALT, AST, GGT, FLI, NFS, FIB-4, and APRI. The weighted Cox proportional risk regression models were utilized to calculate hazard ratios (HRs) and 95% confidence intervals (CIs) for the impact of the dietary quality indexes, considered as both continuous and categorical variables, on all-cause and cardiovascular mortality among participants with NAFLD, adjusting for age, sex, race, BMI, hypertension, and diabetes. What’s more, Cox proportional risk regression models with restricted cubic splines were utilized to determine the nonlinear relationships between these five dietary quality indexes and mortality. In addition, the association between advanced fibrosis and mortality in NAFLD patients was also explored by Cox proportional risk regression models. Then, we examined the influence of five dietary quality indexes on the survival of patients with advanced fibrosis. Finally, we performed subgroup analysis in both NAFLD patients and advanced fibrosis patients by age, sex, race, BMI, hypertension, and diabetes.

We conducted our statistical analyses by employing R version 4.3.3 from the R Foundation^1^. Statistical significance was defined as a two-tailed *p*-value <0.05.

## 3 Results

### 3.1 Baseline characteristics of participants

According to Table 1 and Supplementary Tables S1–S5, a total of 3,634 participants with NAFLD were included in this study. The participants had a mean (SE) age of 50.49 (0.41) years, with 56.09% being male and 71.69% identified as non-Hispanic White. The mean (SE) BMI was 34.60 (0.14) kg/m^2^, and the mean (SE) waist circumference was 114.00 (0.33) cm. Among the participants, 16.84% had hypertension, and 24.79% had diabetes. Additionally, the means (SEs) of aMED, HEI-2020, DASH, AHEI, and DII were 5.79 (0.02), 49.32 (0.25), 26.60 (0.06), 38.14 (0.24), and 1.14 (0.04), respectively.

**Table 1:** Baseline characteristics of NAFLD patients.

Characteristic	Total (*n* = 3,634)
Age (years)	50.49 (0.41)
Sex	
Male	2,021 (56.09)
Female	1,613 (43.91)
Race	
Non-Hispanic Black	700 (9.44)
Non-Hispanic White	1,768 (71.69)
Mexican American	610 (8.58)
Other Hispanic	346 (5.17)
Other race	210 (5.13)
BMI (kg/m^2^)	34.60 (0.14)
BMI	
<25 (kg/m^2^)	36 (0.91)
25 to <30 (kg/m^2^)	799 (21.95)
≥30 (kg/m^2^)	2,799 (77.14)
Waist circumference (cm)	114.00 (0.33)
Hypertension	
Yes	727 (16.84)
No	2,907 (83.16)
Diabetes	
Yes	1,101 (24.79)
No	2,533 (75.21)
AST (U/L)	25.55 (0.35)
ALT (U/L)	28.94 (0.42)
GGT (U/L)	35.12 (0.91)
GHB (%)	5.85 (0.02)
GLU (mmol/L)	6.36 (0.05)
HDL (mmol/L)	1.22 (0.01)
LDL (mmol/L)	3.08 (0.02)
TC (mmol/L)	5.10 (0.03)
TG (mmol/L)	1.80 (0.03)
Platelet (1,000 cells/uL)	249.38 (1.60)
aMED	5.79 (0.02)
aMED	
T1	996 (29.28)
T2	1,488 (39.95)
T3	1,150 (30.78)
HEI-2020	49.32 (0.25)
HEI-2020	
T1	1,211 (34.45)
T2	1,212 (33.22)
T3	1,211 (32.34)
DASH	26.60 (0.06)
DASH	
T1	1,172 (32.52)
T2	1,242 (34.01)
T3	1,220 (33.47)
AHEI	38.14 (0.24)
AHEI	
T1	1,211 (31.52)
T2	1,212 (33.83)
T3	1,211 (34.65)
DII	1.14 (0.04)
DII	
T1	1,211 (36.12)
T2	1,212 (32.98)
T3	1,211 (30.90)

Continuous variables were expressed as weighted means and SEs. Categorical variables were expressed as unweighted number and weighted percent of the participants.

### 3.2 Relationships among dietary quality indexes and their associations with liver function markers

**Figure 2:**
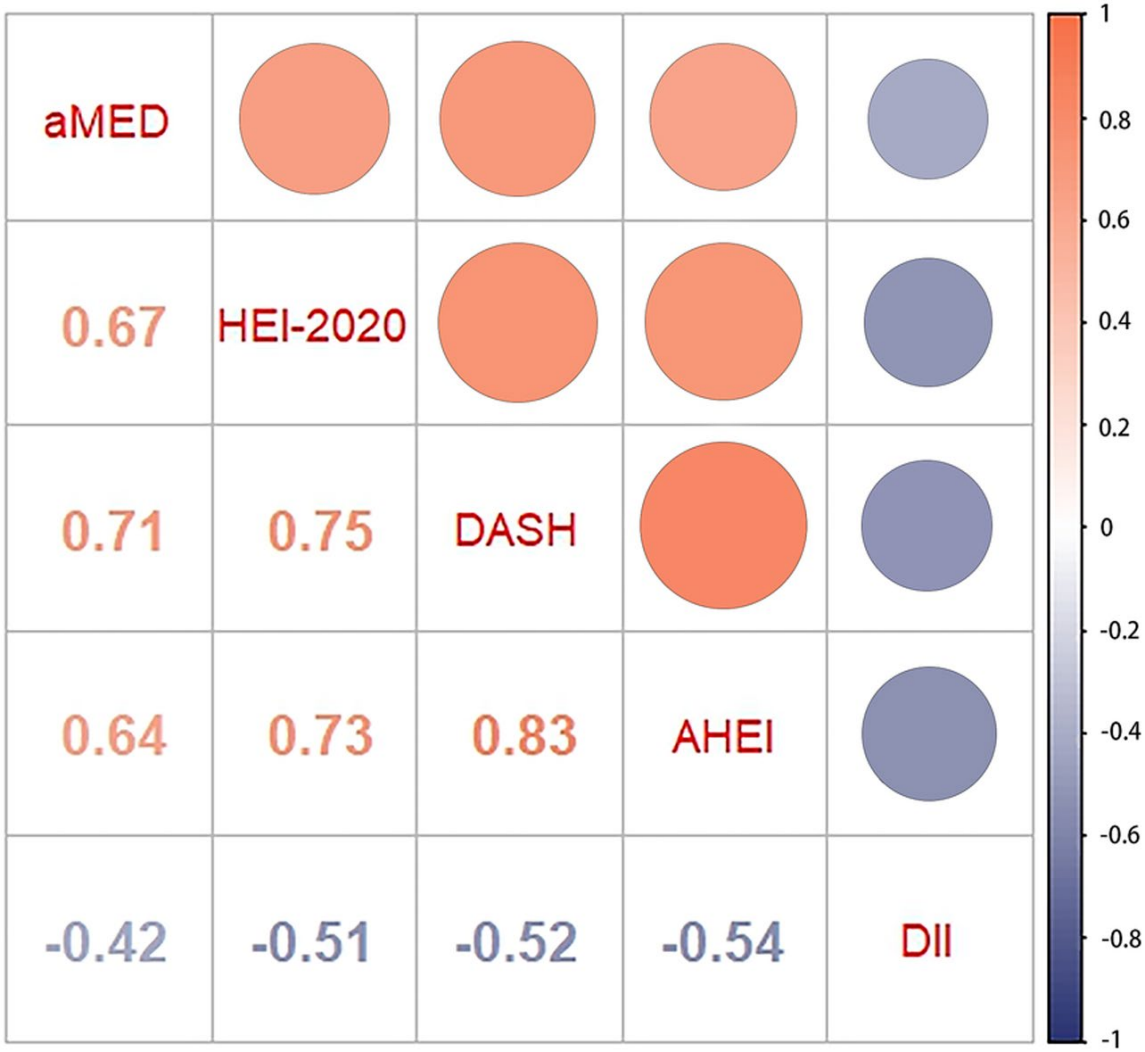
Pairwise correlations between five dietary quality indexes.

As shown in Figure 2, the correlation analysis indicated significant interrelations among all five dietary quality indexes (all *p* values <0.05). The four dietary quality indexes, aMED, HEI-2020, AHEI, and DASH, were positively correlated with each other. Conversely, the DII exhibited negative correlations with these four indexes. We further examined the relationships between these five dietary quality indexes and various liver function markers applying multivariate linear regression, and the results are presented in Tables 2, 3. Notably, aMED, HEI-2020, AHEI, and DASH displayed linear negative correlations with GGT (regression coefficients were − 2.297, −0.222, −0.803, and − 0.167, respectively, and all *p* values <0.05), while DII demonstrated a positive linear association with GGT (regression coefficient = 0.959, *p* = 0.05). In addition, aMED, HEI-2020, AHEI, and DASH were also linearly and negatively associated with FLI (regression coefficients were − 0.530, −0.056, −0.151, and − 0.053, respectively, and all p values <0.05). No linear relationships were observed between the dietary quality indexes and the remaining liver function markers.

**Table 2:** Relationships between five dietary indexes and AST, ALT and GGT.

Variables	AST		ALT		GGT	
	Coeffcient	*p*-value	Coeffcient	*p*-value	Coeffcient	*p*-value
aMED	−0.002	0.995	0.211	0.593	−2.297	0.015
HEI-2020	−0.022	0.608	−0.010	0.763	−0.222	0.004
DASH	−0.037	0.768	−0.005	0.974	−0.803	0.005
AHEI	−0.021	0.590	0.008	0.776	−0.167	0.012
DII	0.113	0.602	0.005	0.979	0.959	0.023

Multivariate linear regression models adjusted for age, sex, race, BMI, hypertension, diabetes.

**Table 3:** Relationships between five dietary indexes and FLI, NFS, FIB-4 and APRI.

Variables	FLI		NFS		FIB-4		APRI	
	Coeffcient	*p*-value	Coeffcient	*p*-value	Coeffcient	*p*-value	Coeffcient	*p*-value
aMED	−0.530	0.009	−0.003	0.911	0.006	0.577	0.000	0.954
HEI-2020	−0.056	<0.001	−0.002	0.356	0.000	0.829	0.000	0.478
DASH	−0.151	0.027	−0.005	0.475	−0.003	0.337	0.000	0.356
AHEI	−0.053	0.002	−0.001	0.562	−0.001	0.447	0.000	0.482
DII	0.198	0.129	−0.010	0.468	0.008	0.323	0.000	0.572

Multivariate linear regression models were adjusted for age, sex, race, BMI, hypertension and diabetes.

### 3.3 Associations of five dietary quality indexes and advanced fibrosis with all-cause and cardiovascular mortality in patients with NAFLD

After a median follow-up of 89 months, 377 out of 3,634 participants with NAFLD had died, with 96 deaths attributed to cardiovascular causes. The results presented in Table 4 indicated that higher scores on the aMED (adjusted HR 0.814, 95% CI 0.681–0.972, *p* = 0.023), HEI-2020 (adjusted HR 0.984, 95% CI 0.972–0.997, *p* = 0.015), DASH (adjusted HR 0.930, 95% CI 0.883–0.979, *p* = 0.006), and AHEI (adjusted HR 0.980, 95% CI 0.966–0.995, *p* = 0.007) were related with lower all-cause mortality, whereas per 1 score increment of DII could increase the risk of all-cause mortality by 23.2% (adjusted HR 1.232, 95% CI 1.136–1.336, *p* < 0.001). Regarding cardiovascular mortality, higher scores on the aMED (adjusted HR 0.719, 95% CI 0.521–0.991, *p* = 0.044), DASH (adjusted HR 0.904, 95% CI 0.834–0.981, *p* = 0.015), and AHEI (adjusted HR 0.976, 95% CI 0.953–1.000, *p* = 0.049) scores were linked to lower cardiovascular mortality. Similar to all-cause mortality findings, a higher DII (adjusted HR 1.280, 95% CI 1.098–1.493, *p* = 0.002) score corresponded to an elevated cardiovascular mortality rate.

**Table 4:** Associations of five dietary quality indexes with all-cause and cardiovascular mortality in patients with NAFLD.

Characteristic	All-cause mortality			Cardiovascular mortality		
	HR	95% CI	*p*-value	HR	95% CI	*p*-value
aMED	0.814	0.681, 0.972	0.023	0.719	0.521, 0.991	0.044
aMED						
T1	–	–		–	–	
T2	0.838	0.564, 1.247	0.384	0.519	0.225, 1.196	0.123
T3	0.565	0.374, 0.853	0.007	0.355	0.160, 0.787	0.011
HEI-2020	0.984	0.972, 0.997	0.015	0.983	0.962, 1.004	0.110
HEI-2020						
T1	–	–		–	–	
T2	0.758	0.593, 0.968	0.026	0.662	0.351, 1.249	0.203
T3	0.675	0.478, 0.953	0.025	0.746	0.322, 1.729	0.495
DASH	0.930	0.883, 0.979	0.006	0.904	0.834, 0.981	0.015
DASH						
T1	–	–		–	–	
T2	0.815	0.561, 1.183	0.282	0.746	0.299, 1.860	0.529
T3	0.619	0.448, 0.854	0.004	0.502	0.268, 0.940	0.031
AHEI	0.980	0.966, 0.995	0.007	0.976	0.953, 1.000	0.049
AHEI						
T1	–	–		–	–	
T2	0.783	0.576, 1.063	0.117	0.894	0.405, 1.974	0.782
T3	0.569	0.412, 0.787	<0.001	0.599	0.300, 1.195	0.146
DII	1.232	1.136, 1.336	<0.001	1.280	1.098, 1.493	0.002
DII						
T1	–	–		–	–	
T2	1.305	0.896, 1.901	0.165	1.261	0.528, 3.014	0.601
T3	2.055	1.444, 2.926	<0.001	2.334	1.015, 5.369	0.046

Cox proportional risk regression models were adjusted for age, sex, race, BMI, hypertension and diabetes.

**Figure 3:**
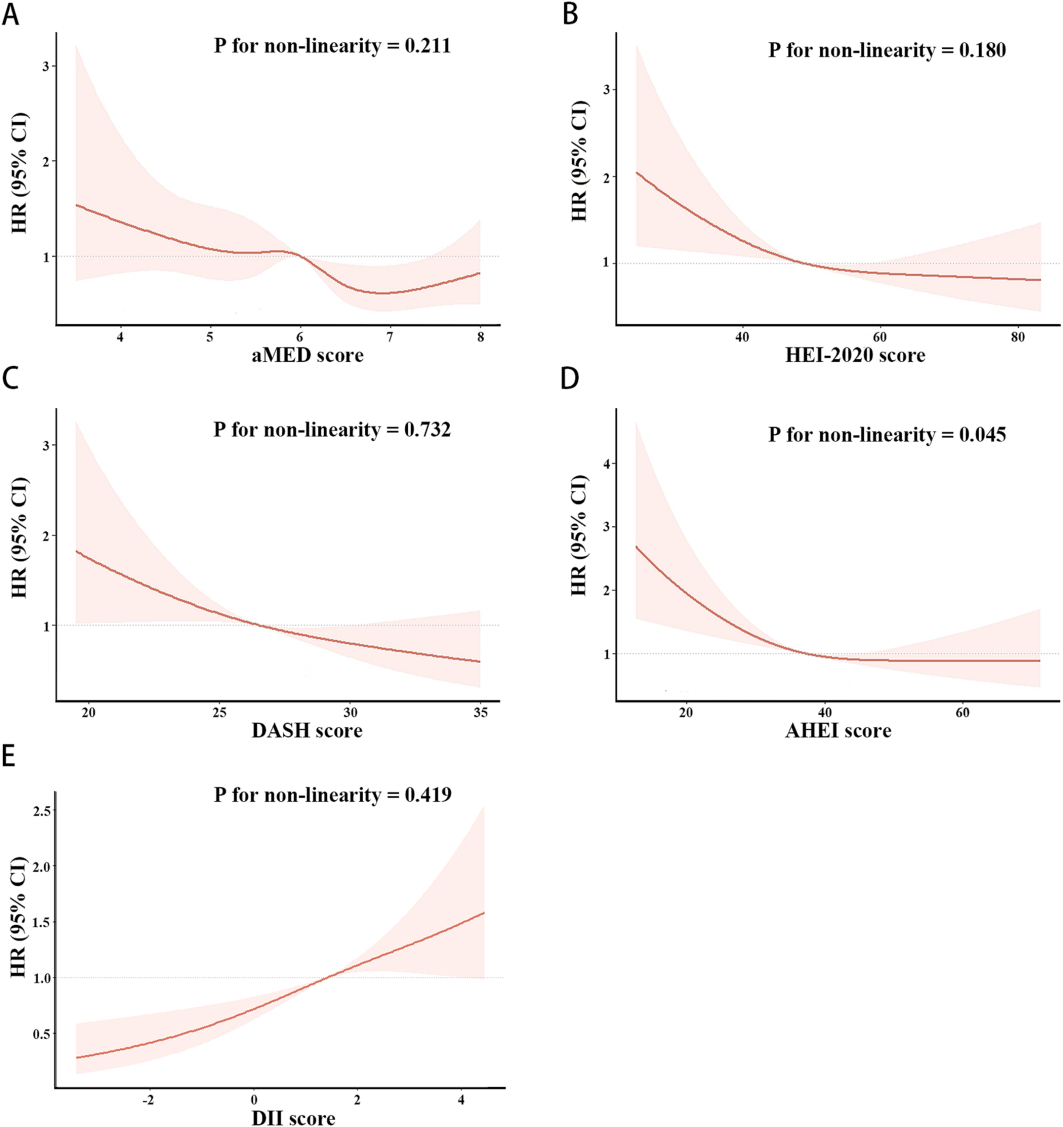
Restricted cubic splines of the associations of aMED **(A)**, HEI-2020 **(B)**, DASH **(C)**, AHEI **(D)**, DII **(E)** with all-cause mortality in patient with NAFLD. HRs (solid lines) and 95% CIs (shaded areas) were adjusted for age, sex, race, BMI, hypertension and diabetes.

**Figure 4:**
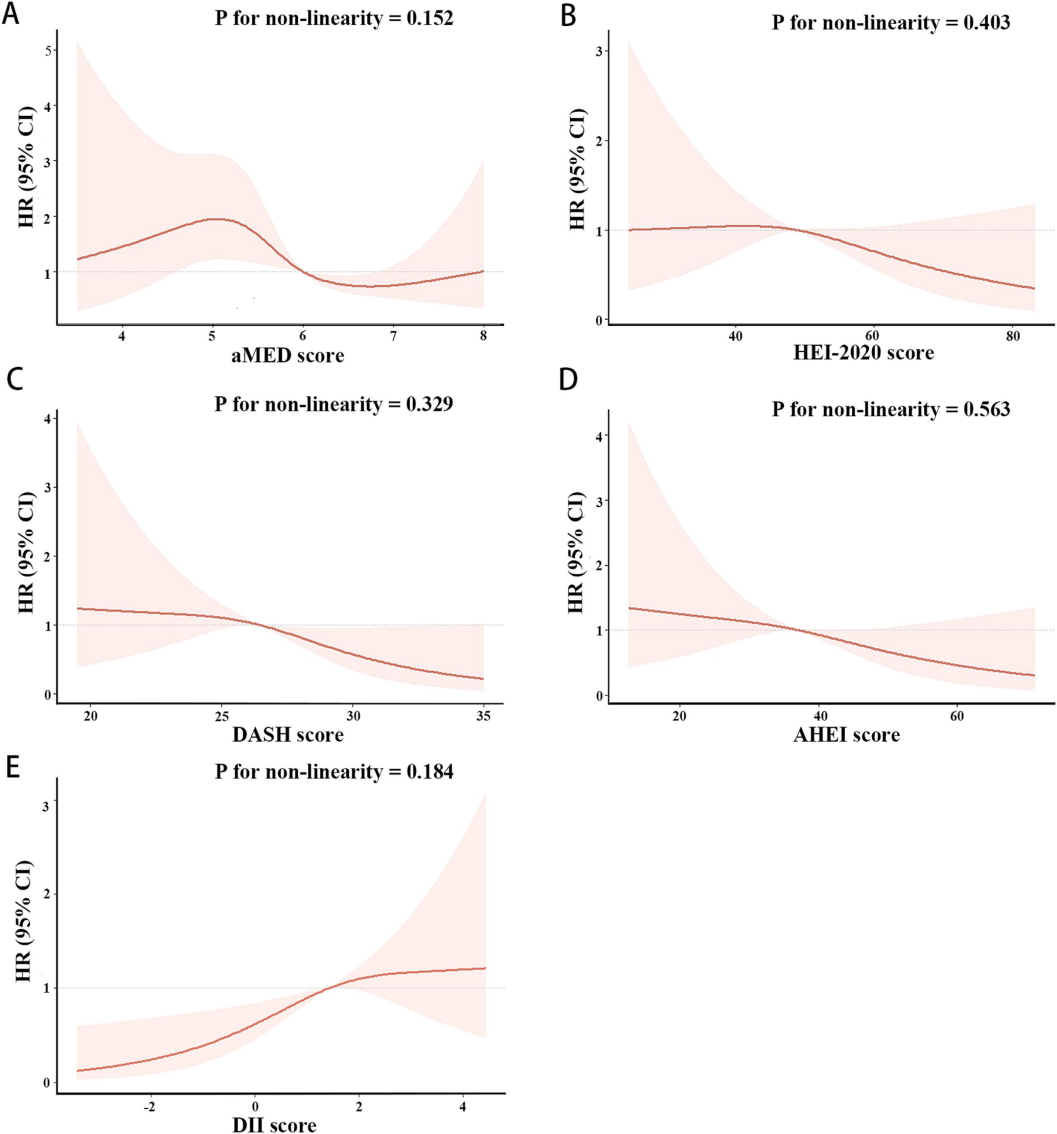
Restricted cubic splines of the associations of aMED **(A)**, HEI-2020 **(B)**, DASH **(C)**, AHEI **(D)**, DII **(E)** with cardiovascular mortality in patient with NAFLD. HRs (solid lines) and 95% CIs (shaded areas) were adjusted for age, sex, race, BMI, hypertension and diabetes.

As shown in Figures 3, 4, the associations of these five dietary quality indexes with all-cause and cardiovascular mortality were thoroughly investigated by restricting cubic splines. We found that only the AHEI had a nonlinear relationship with all-cause mortality in NAFLD patients (p for non-linearity = 0.012).

Our research delved into the impact of advanced fibrosis on all-cause and cardiovascular mortality among NAFLD patients. After adjusting for covariates, NAFLD patients with advanced fibrosis exhibited a 1.639 (95% CI 0.521–0.991, *p* = 0.045)-fold higher risk of all-cause mortality compared to those without advanced fibrosis (Table 5).

**Table 5:** Association of advanced fibrosis with all-cause and cardiovascular mortality in patients with NAFLD.

Characteristic	All-cause mortality			Cardiovascular mortality		
	HR	95% CI	*p*-value	HR	95% CI	*p*-value
Advance fibrosis						
No	–	–		–	–	
Yes	1.639	1.178, 2.280	0.003	1.742	0.956, 3.175	0.070

Cox proportional risk regression models were adjusted for age, sex, race, BMI, hypertension and diabetes.

### 3.4 Associations of dietary quality indexes with mortality in patients with advanced fibrosis

We further explored the associations between five dietary quality indexes and all-cause mortality in patients with advanced fibrosis. Adjusting for covariates, the Cox regression analysis in Table 6 showed that when HEI-2020 was considered as a categorical variable, individuals in the highest tertile (T3) of HEI-2020 scores had significantly lower mortality rates compared to those in the lowest tertile (T1). For DASH, when considered as a continuous variable, each unit increase in DASH score was linked to a 0.921 fold reduction in mortality risk. AHEI was associated with reduced mortality rates in advanced fibrosis participants, both as a continuous variable (adjusted HR 0.971, 95% CI 0.945–0.997, *p* = 0.030) and categorized (T2: adjusted HR 0.545, 95% CI 0.310–0.960, *p* = 0.036; T3: adjusted HR 0.444, 95% CI 0.245–0.804, *p* = 0.007). In contrast, DII, whether as a continuous or categorical variable, was both associated with higher mortality rates in advanced fibrosis individuals (continuous: adjusted HR 1.311, 95% CI 1.121–1.534, *p* = 0.001; T3: adjusted HR 2.772, 95% CI 1.477–5.202, *p* = 0.002).

**Table 6:** Associations of five dietary quality indexes with all-cause mortality in patients with advanced fibrosis.

Characteristic	HR	95% CI	*p*-value
aMED	0.798	0.614, 1.039	0.093
aMED			
T1	–	–	
T2	0.664	0.365, 1.209	0.181
T3	0.558	0.309, 1.010	0.054
HEI-2020	0.978	0.956, 1.001	0.057
HEI-2020			
T1	–	–	
T2	0.821	0.509, 1.324	0.419
T3	0.519	0.280, 0.964	0.038
DASH	0.921	0.849, 0.999	0.048
DASH			
T1	–	–	
T2	0.601	0.329, 1.097	0.097
T3	0.627	0.368, 1.069	0.086
AHEI	0.971	0.945, 0.997	0.030
AHEI			
T1	–	–	
T2	0.545	0.310, 0.960	0.036
T3	0.444	0.245, 0.804	0.007
DII	1.311	1.121, 1.534	0.001
DII			
T1	–	–	
T2	1.606	0.894, 2.884	0.113
T3	2.772	1.477, 5.202	0.002

Cox proportional risk regression models were adjusted for age, sex, race, BMI, hypertension and diabetes.

### 3.5 Subgroup analysis

**Figure 5:**
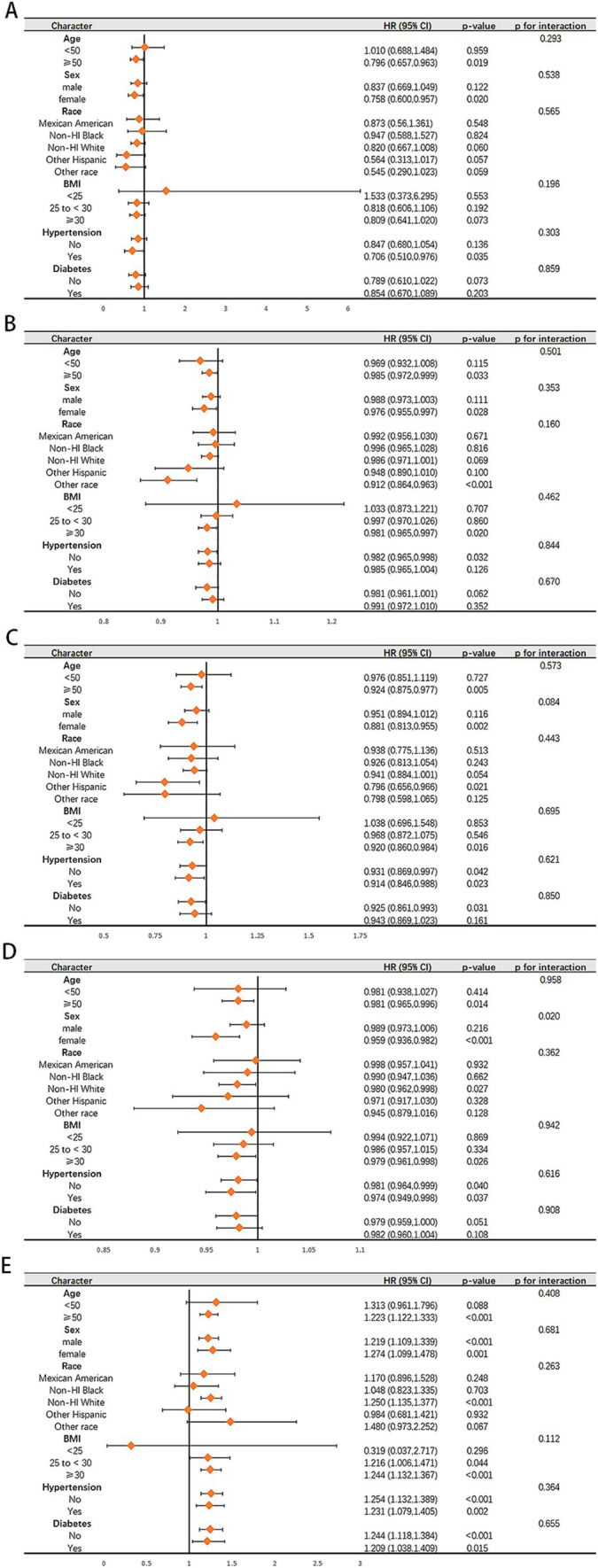
Subgroup analysis of the associations of aMED **(A)**, HEI-2020 **(B)**, DASH **(C)**, AHEI **(D)**, DII **(E)** with all-cause in patient with NAFLD. Adjusted for age, sex, race, BMI, hypertension and diabetes.

The results of subgroup analysis of the connections between five dietary quality indexes and all-cause mortality in the NAFLD population and the advanced fibrosis population are shown in Figure 5 and Supplementary Table S6. We only found an interaction between AHEI score and sex in terms of survival for patients with NAFLD (p for interaction = 0.020). Higher AHEI scores were observed to be significantly linked to lower all-cause mortality in women (adjusted HR 0.959, 95% CI 0.936–0.982, *p* < 0.001), while no such association was observed in men (adjusted HR 0.989, 95% CI 0.973–1.006, *p* = 0.216).

## 4 Discussion

In this research, we explored the associations between dietary patterns and mortality rates in a population of 3,634 individuals who fulfilled the diagnostic criteria for NAFLD based on data from the NHANES 2005–2018. Furthermore, we examined the impact of diet on all-cause mortality in patients suffering from advanced liver fibrosis. We observed that the aMED, HEI-2020, DASH, and AHEI dietary scores were positively correlated with each other, indicating that healthier dietary patterns tend to overlap across different indexes. On the other hand, the DII was negatively correlated with these four previous dietary scores, suggesting that a more inflammatory diet may be inversely related to overall dietary quality. Moreover, our investigation of the relationships between five dietary quality indexes and liver enzyme and fibrosis markers revealed that the aMED, HEI-2020, AHEI, and DASH dietary scores exhibited a linear negative correlation with GGT and FLI, while the DII showed a positive correlation with GGT. GGT is a sensitive marker reflecting hepatic fat accumulation and mild hepatocellular damage, often significantly increasing during the early stages of NAFLD (47). Elevated FLI is typically associated with increased hepatic fat deposition and accelerated fibrosis progression (48, 49). These findings suggest that high-quality diets may improve NAFLD prognosis through mechanisms such as reducing liver fat accumulation, potentially slowing the progression of fibrosis. Most importantly, our study found that high-quality dietary patterns were significantly linked to reduced mortality in patients with NAFLD. This association was also evident in patients with advanced liver fibrosis, suggesting that dietary improvements may be particularly beneficial in reducing mortality risk in this high-risk subgroup. Given the growing prevalence of NAFLD and its related complications, these findings highlight the importance of dietary interventions as a modifiable factor that can complement medical management strategies in mitigating the adverse outcomes of NAFLD.

The definitions of the five dietary quality indexes (aMED, HEI-2020, AHEI, DASH, and DII) used in our study have been previously elaborated. The MED, recognized as the most recommended regimen for individuals with NAFLD, involves a plant-based, protein-rich, low-fat diet with minimal processed components. It has been proven to prevent cardiovascular disease and diabetes and to reduce mortality in patients with metabolic syndrome, all of which are highly associated with NAFLD (50–52). The HEI is a dietary index developed to evaluate compliance with the Dietary Guidelines for Americans (20). Utilizing a density-based approach, it calculates the number of foods and nutrients consumed per 1,000 calories of energy intake, emphasizing meeting nutritional requirements primarily through dietary sources rather than supplements. The HEI-2020 encompasses 13 components to evaluate a multidimensional, dynamic dietary pattern. The AHEI was developed by McCullough and colleagues in 2002 as an enhancement to the original HEI, with the aim of forecasting the chronic disease risk associated with adherence to dietary guidelines among Americans. The DASH diet was initially formulated to manage and prevent hypertension (53, 54). Numerous studies have demonstrated that the DASH diet not only addresses hypertension but also influences the occurrence and progression of various other diseases, including NAFLD (55–57). The DII was progressively developed as researchers’ comprehension of inflammation’s role in health and illness evolved (58–60). DII quantifies the inflammatory potential of participants’ diets by assessing six inflammatory biomarkers, including TNF-*α* and C-reactive protein. It allows for the quantification and comparison of dietary inflammation levels among individuals, enabling the exploration of the association between dietary inflammation levels and specific diseases (61–66).

Prior studies consistently validated the associations between these specific dietary patterns and NAFLD. For example, several research studies showed higher adherence to the MED, AHEI, and HEI diets was associated with a reduced likelihood of developing NAFLD (67–69). A randomized controlled trial demonstrated that the DASH diet exerted beneficial effects on liver enzymes, TGs, inflammatory markers, and insulin metabolism in NAFLD individuals, with recent reports further endorsing its potential as a management strategy for these patients (70). Likewise, the dietary inflammatory index was linked to NAFLD and an increased risk of advanced liver fibrosis, as evidenced by a study using data from the NHANES database (71). Additionally, another investigation using NHANES data highlighted significant correlations among these five dietary quality indexes and their associations with the metabolic dysfunction-associated fatty liver disease (MAFLD) phenotype (15). Collectively, these indexes reflect comprehensive dietary habits, and substantial evidence supports their close association with NAFLD development. These findings establish a foundation for our study to utilize these five validated indexes in assessing diet quality.

Previous studies emphasized the pivotal role of diet quality in influencing mortality and disease progression in NAFLD patients, providing valuable insights for interpreting our findings. A recent cross-sectional investigation identified a linear inverse relationship between diet quality and all-cause mortality among NAFLD patients, suggesting that higher dietary quality contributes to improved survival outcomes (72). Another study demonstrated that in MAFLD patients lacking regular physical activity, higher dietary quality (assessed with HEI) exerted a stronger protective effect on reducing all-cause, cardiovascular-related, and cancer-related mortality than physical activity alone (73). This underscores the potential of dietary interventions to improve outcomes in liver diseases, particularly among sedentary populations with limited physical activity. Moreover, patients with elevated DII scores face increased hospitalization rates and mortality risks, highlighting that reducing inflammatory food consumption could help mitigate liver steatosis and fibrosis progression in NAFLD patients (74, 75). Conversely, conflicting evidence exists. A cohort study utilizing NHANES data found that while higher diet quality was associated with a lower risk of all-cause mortality in individuals without NAFLD, no such association was observed in those with NAFLD (16). This discrepancy suggests that the protective effect of diet quality may diminish, possibly due to disease severity, complications, or other confounding factors. These findings highlight the complexity of dietary impacts and underscore the necessity for further research to clarify the conditions under which diet quality influences NAFLD prognosis. On the whole, these above findings reinforce the importance of dietary quality as a modifiable factor in NAFLD management, providing a basis for our study to explore the association between dietary quality and patient outcomes.

The presence of advanced fibrosis poses a risk factor for the progression of NAFLD and increased mortality rates (5, 8, 76). Our study identified that individuals with NAFLD and advanced fibrosis exhibited an independent association with a higher all-cause mortality risk. Similar findings were observed in previous research (77, 78). What’s more, our analysis demonstrated independent and inverse relationships between high-quality dietary patterns and all-cause mortality in NAFLD patients with advanced fibrosis. An previous observational study based on the American Framingham Heart Study and NHANES elucidated the negative association between a healthy diet and liver fat accumulation and fibrosis progression by correlating pre-existing dietary scores with these liver parameters in participants (79). Another investigation into the impact of dietary habits on the association between severe liver fibrosis and mortality rates in the Korean population also identified a similar influence in people with moderate and low-quality diets (80). Our findings corroborate prior research, emphasizing the potential significance of improving dietary patterns for NAFLD patients with advanced fibrosis to enhance survival outcomes and minimize adverse effects.

Our research suggests that adopting beneficial dietary practices may positively impact the mortality risks of individuals with NAFLD and advanced fibrosis. Nevertheless, the precise dietary constituents that confer this benefit and the underlying mechanisms remain elusive. Extensive research showed that certain dietary elements can influence NAFLD outcomes. Expert opinion supports the idea that reducing free sugars, trans fatty acids, saturated fatty acids (SFA), and cholesterol can mitigate fatty liver and fibrosis in NAFLD patients. Free sugars refer to refined sugars added to beverages and processed foods, predominantly sucrose, fructose, and high fructose corn syrup (81). Epidemiological data consistently link excessive added sugar consumption to an increased risk of NAFLD development and worsening liver fibrosis (82, 83). Furthermore, emerging evidence indicates that NASH patients tend to have higher intakes of SFA and cholesterol but lower levels of polyunsaturated fatty acid (PUFA) (84, 85). The recommended MED is not a low-fat diet; on the contrary, 40% of its calorie content is from fat, but it shows a preference for high-quality fats. Olive oil, nuts, and fish are rich sources of MUFA and n-3 PUFA (including eicosapentaenoic acid and docosahexaenoic acid), which can improve glucose metabolism and insulin sensitivity, reduce hepatic TG content, and alleviate fatty hepatitis (86–88). Additionally, a n-3 PUFA-rich diet demonstrated a protective effect against hepatocellular carcinoma (HCC), especially crucial given the rising incidence of HCC in individuals with NAFLD and advanced fibrosis, endorsing the significance of enhancing dietary quality in reducing mortality rates (89). What’s more, polyphenols, abundant in the MED, play a vital role in protecting NAFLD patients by reducing fat accumulation, enhancing fatty acid oxidation, and decreasing oxidative stress to prevent liver cell damage (90). *In vitro* studies suggested that flavonoids within polyphenols may delay the absorption of glucose by inhibiting intestinal glucosidases, thereby reducing postprandial hyperinsulinemia and hyperglycemia (91). Moreover, regardless of specific dietary components, consensus statements recognize that a high-quality, nutritious diet, coupled with caloric restriction and gradual weight loss, leads to improvements in serum liver enzymes, liver fat levels, and fibrosis (92).

The advantages of our investigation lie in the comprehensive array of dietary quality indexes. These indexes offer a superior benefit over individual dietary components and nutrients by capturing the overall diet quality and considering the synergistic interactions among diverse foods. Additionally, the data analyzed in our study was sourced from the NHANES, which provided comprehensive health data that was representative of the entire US population, ensuring the credibility and accuracy of our research. However, our research has several limitations. Firstly, our study is an observational study conducted through the NHANES database. While our findings suggest that a high-quality diet may influence the survival of individuals with NAFLD and those with NAFLD accompanied by advanced fibrosis, further exploration of the precise mechanisms is necessary. Secondly, we identified NAFLD patients using non-invasive biomarkers. Although these markers are less specific than the gold standard liver biopsy for NAFLD diagnosis, existing research substantiates the reliability and effectiveness of non-invasive diagnostic markers (93–95). Thirdly, the method of collecting nutritional status data in NHANES is confined to 24-h dietary recalls, which is susceptible to self-reporting bias and may not be sufficient to comprehensively assess long-term diet quality or reflect individual food preferences. Fourthly, the lack of pertinent information on liver-related mortality in NHANES limits our ability to conduct further analyses. Fifth, although we have controlled covariates such as diabetes and hypertension, the real situation is complex and we may not be able to completely exclude the interference of other confounding factors.

## 5 Conclusion

In conclusion, our observational study of the American population highlights the significant role of dietary quality in reducing mortality risk among NAFLD patients. Notably, while advanced fibrosis is an independent predictor of mortality in this population, our findings suggest that a high-quality diet can mitigate this risk. By demonstrating the beneficial effects of high-quality diets on both NAFLD and advanced fibrosis patients, our research provides theoretical support for integrating dietary improvements into the management of these conditions. We propose that nutritional strategies may serve as an effective, non-pharmacological approach to reducing mortality rates and improving outcomes in individuals with NAFLD and advanced liver fibrosis.

## Data Availability

Publicly available datasets were analyzed in this study. This data can be found at: https://www.cdc.gov/nchs/nhanes/index.htm.

## Footnotes

^1^ http://www.R-project.org
