# Supplementary material for: Association of dietary quality and mortality in the non-alcoholic fatty liver disease and advanced fibrosis populations: NHANES 2005–2018
Source: Front Nutr. 2025 Jan 23;12:1507342. doi: 10.3389/fnut.2025.1507342 (PMC11798782; doi:10.3389/fnut.2025.1507342)
Supplement: Supplementary file 1 [file Table_1.docx]

**Table S1.** Baseline characteristics of NAFLD patients according to the aMED score

| Characteristic | T1 | T2 | T3 | *p*-value |
| --- | --- | --- | --- | --- |
| Age (years) | 46.79 (0.60) | 50.20 (0.53) | 54.37 (0.72) | **<0.001** |
| Sex |  |  |  | 0.221 |
| Male | 583 (59.09) | 816 (55.18) | 622 (54.44) |  |
| Female | 413 (40.91) | 672 (44.82) | 528 (45.56) |  |
| Race |  |  |  | 0.189 |
| Non-Hispanic Black | 187 (9.13) | 306 (10.84) | 207 (7.92) |  |
| Non-Hispanic White | 553 (74.47) | 687 (69.57) | 528 (71.80) |  |
| Mexican American | 131 (7.16) | 264 (9.20) | 215 (9.10) |  |
| Other Hispanic | 78 (4.48) | 153 (5.55) | 115 (5.33) |  |
| Other race | 47 (4.76) | 78 (4.84) | 85 (5.85) |  |
| BMI (kg/m^2^) | 35.07 (0.30) | 34.95 (0.22) | 33.71 (0.21) | **<0.001** |
| BMI |  |  |  | 0.077 |
| <25 (kg/m^2^) | 9 (0.97) | 12 (0.59) | 15 (1.26) |  |
| 25 to < 30 (kg/m^2^) | 196 (19.58) | 313 (21.03) | 290 (25.39) |  |
| ≥30 (kg/m^2^) | 791 (79.45) | 1,163 (78.37) | 845 (73.35) |  |
| Waist circumference (cm) | 115.18 (0.61) | 114.57 (0.53) | 112.14 (0.48) | **<0.001** |
| Hypertension |  |  |  | 0.371 |
| Yes | 181 (16.48) | 289 (15.74) | 257 (18.60) |  |
| No | 815 (83.52) | 1,199 (84.26) | 893 (81.40) |  |
| Diabetes |  |  |  | 0.371 |
| Yes | 276 (22.98) | 452 (24.76) | 373 (26.54) |  |
| No | 720 (77.02) | 1,036 (75.24) | 777 (73.46) |  |
| AST (U/L) | 25.29 (0.72) | 26.13 (0.56) | 25.04 (0.41) | 0.095 |
| ALT (U/L) | 28.84 (0.69) | 30.09 (0.74) | 27.53 (0.63) | 0.142 |
| GGT (U/L) | 37.42 (2.32) | 35.61 (1.35) | 32.28 (1.11) | 0.191 |
| GHB (%) | 5.85 (0.05) | 5.87 (0.03) | 5.84 (0.04) | 0.436 |
| GLU(mmol/L) | 6.35 (0.10) | 6.39 (0.07) | 6.33 (0.08) | 0.251 |
| HDL (mmol/L) | 1.22 (0.02) | 1.22 (0.01) | 1.24 (0.01) | 0.482 |
| LDL (mmol/L) | 3.17 (0.05) | 3.06 (0.04) | 3.01 (0.04) | 0.073 |
| TC (mmol/L) | 5.19 (0.06) | 5.06 (0.05) | 5.06 (0.05) | 0.250 |
| TG (mmol/L) | 1.83 (0.04) | 1.75 (0.04) | 1.81 (0.05) | 0.603 |
| Platelet (1000 cells/uL) | 255.08 (2.83) | 247.97 (2.53) | 245.79 (3.07) | **0.037** |
| HEI-2020 | 40.87 (0.44) | 48.44 (0.26) | 58.50 (0.41) | **<0.001** |
| HEI-2020 |  |  |  | **<0.001** |
| T1 | 668 (67.16) | 461 (31.17) | 82 (7.58) |  |
| T2 | 273 (27.84) | 635 (42.27) | 304 (26.58) |  |
| T3 | 55 (5.00) | 392 (26.56) | 764 (65.83) |  |
| DASH | 24.29 (0.10) | 26.47 (0.07) | 28.97 (0.10) | **<0.001** |
| DASH |  |  |  | **<0.001** |
| T1 | 688 (68.01) | 433 (28.38) | 51 (4.15) |  |
| T2 | 266 (27.89) | 654 (43.46) | 322 (27.57) |  |
| T3 | 42 (4.10) | 401 (28.17) | 777 (68.28) |  |
| AHEI | 30.10 (0.40) | 37.82 (0.29) | 46.19 (0.43) | **<0.001** |
| AHEI |  |  |  | **<0.001** |
| T1 | 639 (63.15) | 469 (27.45) | 103 (6.70) |  |
| T2 | 294 (29.93) | 597 (41.11) | 321 (28.10) |  |
| T3 | 63 (6.92) | 422 (31.44) | 726 (65.20) |  |
| DII | 1.97 (0.07) | 1.20 (0.05) | 0.27 (0.07) | **<0.001** |
| DII |  |  |  | **<0.001** |
| T1 | 146 (16.80) | 437 (33.79) | 628 (57.53) |  |
| T2 | 330 (34.48) | 537 (35.25) | 345 (28.60) |  |
| T3 | 520 (48.71) | 514 (30.95) | 177 (13.87) |  |

Continuous variables were expressed as weighted means (SEs), and *p*-values are derived using the Student’s t-test. Categorical variables were expressed as unweighted number (weighted percent), and *p*-values are derived using the chi-square test.
