# Supplementary material for: Association of dietary quality and mortality in the non-alcoholic fatty liver disease and advanced fibrosis populations: NHANES 2005–2018
Source: Front Nutr. 2025 Jan 23;12:1507342. doi: 10.3389/fnut.2025.1507342 (PMC11798782; doi:10.3389/fnut.2025.1507342)
Supplement: Supplementary file 2 [file Table_2.docx]

**Table S2.** Baseline characteristics of NAFLD patients according to the HEI-2020 score

| Characteristic | T1 | T2 | T3 | *p*-value |
| --- | --- | --- | --- | --- |
| Age (years) | 46.85 (0.58) | 50.92 (0.62) | 53.91 (0.63) | **<0.001** |
| Sex |  |  |  | 0.139 |
| Male | 526 (44.25) | 514 (40.64) | 573 (46.89) |  |
| Female | 685 (55.75) | 698 (59.36) | 638 (53.11) |  |
| Race |  |  |  | 0.199 |
| Non-Hispanic Black | 178 (8.86) | 200 (7.91) | 232 (8.95) |  |
| Non-Hispanic White | 255 (10.70) | 243 (9.95) | 202 (7.57) |  |
| Mexican American | 627 (71.01) | 588 (72.76) | 553 (71.32) |  |
| Other Hispanic | 91 (4.66) | 109 (4.38) | 146 (6.51) |  |
| Other race | 60 (4.77) | 72 (5.00) | 78 (5.64) |  |
| BMI (kg/m^2^) | 35.39 (0.26) | 34.46 (0.26) | 33.91 (0.22) | **<0.001** |
| BMI |  |  |  | 0.078 |
| <25 (kg/m^2^) | 12 (0.86) | 12 (0.97) | 12 (0.90) |  |
| 25 to < 30 (kg/m^2^) | 962 (80.81) | 932 (75.54) | 905 (74.88) |  |
| ≥30 (kg/m^2^) | 237 (18.33) | 268 (23.49) | 294 (24.22) |  |
| Waist circumference (cm) | 115.42 (0.50) | 114.28 (0.62) | 112.19 (0.49) | **<0.001** |
| Hypertension |  |  |  | **0.048** |
| Yes | 230 (15.22) | 229 (15.60) | 268 (19.83) |  |
| No | 981 (84.78) | 983 (84.40) | 943 (80.17) |  |
| Diabetes |  |  |  | 0.060 |
| Yes | 875 (78.26) | 860 (74.62) | 798 (72.57) |  |
| No | 336 (21.74) | 352 (25.38) | 413 (27.43) |  |
| AST (U/L) | 25.92 (0.79) | 25.04 (0.38) | 25.67 (0.43) | **0.038** |
| ALT (U/L) | 29.75 (0.84) | 28.75 (0.64) | 28.26 (0.55) | 0.884 |
| GGT (U/L) | 37.86 (1.96) | 34.96 (1.44) | 32.35 (1.00) | 0.175 |
| GHB (%) | 5.85 (0.04) | 5.87 (0.04) | 5.84 (0.04) | 0.554 |
| GLU(mmol/L) | 6.32 (0.07) | 6.40 (0.09) | 6.36 (0.09) | 0.109 |
| HDL (mmol/L) | 1.21 (0.01) | 1.23 (0.01) | 1.23 (0.01) | 0.226 |
| LDL (mmol/L) | 3.10 (0.04) | 3.09 (0.04) | 3.04 (0.04) | 0.769 |
| TC (mmol/L) | 5.09 (0.05) | 5.12 (0.05) | 5.08 (0.05) | 0.786 |
| TG (mmol/L) | 1.78 (0.04) | 1.80 (0.04) | 1.81 (0.05) | 0.798 |
| Platelet (1000 cells/uL) | 253.28 (2.29) | 249.88 (2.90) | 244.71 (3.06) | 0.112 |
| aMED | 5.17 (0.03) | 5.76 (0.03) | 6.48 (0.03) | **<0.001** |
| aMED |  |  |  | **<0.001** |
| T1 | 668 (57.08) | 273 (24.54) | 55 (4.53) |  |
| T2 | 461 (36.15) | 635 (50.83) | 392 (32.81) |  |
| T3 | 82 (6.77) | 304 (24.63) | 764 (62.66) |  |
| DASH | 24.51 (0.09) | 26.52 (0.08) | 28.91 (0.09) | **<0.001** |
| DASH |  |  |  | **<0.001** |
| T1 | 804 (65.14) | 318 (26.16) | 50 (4.31) |  |
| T2 | 339 (28.01) | 605 (48.81) | 298 (25.20) |  |
| T3 | 68 (6.85) | 289 (25.03) | 863 (70.49) |  |
| AHEI | 30.26 (0.34) | 37.83 (0.32) | 46.84 (0.32) | **<0.001** |
| AHEI |  |  |  | **<0.001** |
| T1 | 799 (62.69) | 343 (25.48) | 69 (4.51) |  |
| T2 | 334 (29.99) | 557 (44.42) | 321 (27.04) |  |
| T3 | 78 (7.32) | 312 (30.09) | 821 (68.45) |  |
| DII | 2.03 (0.07) | 1.16 (0.06) | 0.17 (0.07) | **<0.001** |
| DII |  |  |  | **<0.001** |
| T1 | 157 (14.47) | 375 (35.01) | 679 (60.33) |  |
| T2 | 383 (34.69) | 477 (37.98) | 352 (26.01) |  |
| T3 | 671 (50.84) | 360 (27.00) | 180 (13.65) |  |

Continuous variables were expressed as weighted means (SEs), and *p*-values are derived using the Student’s t-test. Categorical variables were expressed as unweighted number (weighted percent), and *p*-values are derived using the chi-square test.
