# Supplementary material for: Association of dietary quality and mortality in the non-alcoholic fatty liver disease and advanced fibrosis populations: NHANES 2005–2018
Source: Front Nutr. 2025 Jan 23;12:1507342. doi: 10.3389/fnut.2025.1507342 (PMC11798782; doi:10.3389/fnut.2025.1507342)
Supplement: Supplementary file 3 [file Table_3.docx]

**Table S3.** Baseline characteristics of NAFLD patients according to the DASH score

| Characteristic | T1 | T2 | T3 | *p*-value |
| --- | --- | --- | --- | --- |
| Age (years) | 46.74 (0.57) | 51.14 (0.54) | 53.47 (0.67) | **<0.001** |
| Sex |  |  |  | 0.073 |
| Male | 692 (58.80) | 659 (52.68) | 670 (56.93) |  |
| Female | 480 (41.20) | 583 (47.32) | 550 (43.07) |  |
| Race |  |  |  | 0.080 |
| Non-Hispanic Black | 260 (10.86) | 259 (10.44) | 181 (7.04) |  |
| Non-Hispanic White | 607 (70.92) | 590 (72.36) | 571 (71.77) |  |
| Mexican American | 142 (7.57) | 211 (8.86) | 257 (9.27) |  |
| Other Hispanic | 92 (5.19) | 125 (4.42) | 129 (5.92) |  |
| Other race | 71 (5.47) | 57 (3.92) | 82 (6.01) |  |
| BMI (kg/m^2^) | 35.33 (0.26) | 34.79 (0.24) | 33.71 (0.22) | **<0.001** |
| BMI |  |  |  | **0.003** |
| <25 (kg/m^2^) | 14 (1.10) | 10 (0.59) | 12 (1.05) |  |
| 25 to < 30 (kg/m^2^) | 222 (18.01) | 259 (20.92) | 318 (26.82) |  |
| ≥30 (kg/m^2^) | 936 (80.89) | 973 (78.49) | 890 (72.13) |  |
| Waist circumference (cm) | 115.48 (0.54) | 114.43 (0.58) | 112.12 (0.49) | **<0.001** |
| Hypertension |  |  |  | 0.472 |
| Yes | 223 (15.51) | 231 (16.72) | 273 (18.25) |  |
| No | 949 (84.49) | 1,011 (83.28) | 947 (81.75) |  |
| Diabetes |  |  |  | 0.887 |
| Yes | 355 (24.98) | 376 (25.24) | 370 (24.14) |  |
| No | 817 (75.02) | 866 (74.76) | 850 (75.86) |  |
| AST (U/L) | 26.02 (0.87) | 25.10 (0.47) | 25.55 (0.41) | **0.021** |
| ALT (U/L) | 29.85 (0.93) | 28.70 (0.73) | 28.28 (0.62) | 0.709 |
| GGT (U/L) | 36.85 (1.84) | 35.76 (1.70) | 32.78 (1.07) | 0.583 |
| GHB (%) | 5.88 (0.04) | 5.86 (0.04) | 5.82 (0.04) | 0.787 |
| GLU(mmol/L) | 6.37 (0.09) | 6.40 (0.08) | 6.31 (0.09) | 0.763 |
| HDL (mmol/L) | 1.19 (0.01) | 1.25 (0.01) | 1.24 (0.01) | **<0.001** |
| LDL (mmol/L) | 3.06 (0.04) | 3.10 (0.04) | 3.07 (0.04) | 0.720 |
| TC (mmol/L) | 5.03 (0.05) | 5.13 (0.05) | 5.13 (0.05) | 0.105 |
| TG (mmol/L) | 1.79 (0.03) | 1.77 (0.04) | 1.83 (0.05) | 0.468 |
| Platelet (1000 cells/uL) | 250.43 (2.70) | 249.33 (2.73) | 248.41 (2.90) | 0.752 |
| aMED | 5.09 (0.02) | 5.77 (0.03) | 6.50 (0.03) | **<0.001** |
| aMED |  |  |  | **<0.001** |
| T1 | 688 (61.22) | 266 (24.01) | 42 (3.59) |  |
| T2 | 433 (34.86) | 654 (51.04) | 401 (33.62) |  |
| T3 | 51 (3.92) | 322 (24.95) | 777 (62.79) |  |
| HEI-2020 | 40.59 (0.34) | 48.67 (0.35) | 58.46 (0.43) | **<0.001** |
| HEI-2020 |  |  |  | **<0.001** |
| T1 | 804 (69.00) | 339 (28.37) | 68 (7.05) |  |
| T2 | 318 (26.72) | 605 (47.67) | 289 (24.84) |  |
| T3 | 50 (4.29) | 298 (23.96) | 863 (68.11) |  |
| AHEI | 28.63 (0.29) | 37.62 (0.26) | 47.90 (0.34) | **<0.001** |
| AHEI |  |  |  | **<0.001** |
| T1 | 859 (71.40) | 311 (22.22) | 41 (2.21) |  |
| T2 | 285 (25.47) | 646 (53.44) | 281 (22.03) |  |
| T3 | 28 (3.13) | 285 (24.34) | 898 (75.76) |  |
| DII | 2.05 (0.06) | 1.26 (0.07) | 0.14 (0.06) | **<0.001** |
| DII |  |  |  | **<0.001** |
| T1 | 155 (14.20) | 349 (31.99) | 707 (61.63) |  |
| T2 | 386 (35.34) | 469 (36.10) | 357 (27.52) |  |
| T3 | 631 (50.46) | 424 (31.92) | 156 (10.85) |  |

Continuous variables were expressed as weighted means (SEs), and *p*-values are derived using the Student’s t-test. Categorical variables were expressed as unweighted number (weighted percent), and *p*-values are derived using the chi-square test.
