# Supplementary material for: Association of dietary quality and mortality in the non-alcoholic fatty liver disease and advanced fibrosis populations: NHANES 2005–2018
Source: Front Nutr. 2025 Jan 23;12:1507342. doi: 10.3389/fnut.2025.1507342 (PMC11798782; doi:10.3389/fnut.2025.1507342)
Supplement: Supplementary file 4 [file Table_4.docx]

**Table S4.** Baseline characteristics of NAFLD patients according to the AHEI score

| Characteristic | T1 | T2 | T3 | *p*-value |
| --- | --- | --- | --- | --- |
| Age (years) | 46.32 (0.66) | 51.29 (0.63) | 53.49 (0.62) | **<0.001** |
| Sex |  |  |  | 0.431 |
| Male | 692 (56.19) | 644 (54.24) | 685 (57.82) |  |
| Female | 519 (43.81) | 568 (45.76) | 526 (42.18) |  |
| Race |  |  |  | **0.013** |
| Non-Hispanic Black | 280 (12.85) | 227 (8.67) | 193 (7.09) |  |
| Non-Hispanic White | 580 (67.26) | 585 (72.62) | 603 (74.81) |  |
| Mexican American | 169 (9.01) | 216 (8.65) | 225 (8.10) |  |
| Other Hispanic | 123 (6.34) | 115 (4.60) | 108 (4.65) |  |
| Other race | 59 (4.53) | 69 (5.46) | 82 (5.35) |  |
| BMI (kg/m^2^) | 35.18 (0.26) | 34.64 (0.24) | 34.03 (0.23) | **0.005** |
| BMI |  |  |  | 0.079 |
| <25 (kg/m^2^) | 13 (1.01) | 12 (0.91) | 11 (0.82) |  |
| 25 to < 30 (kg/m^2^) | 247 (19.44) | 245 (20.41) | 307 (25.74) |  |
| ≥30 (kg/m^2^) | 951 (79.56) | 955 (78.68) | 893 (73.45) |  |
| Waist circumference (cm) | 115.07 (0.54) | 114.11 (0.54) | 112.92 (0.52) | **0.019** |
| Hypertension |  |  |  | 0.327 |
| Yes | 228 (15.28) | 230 (16.52) | 269 (18.56) |  |
| No | 983 (84.72) | 982 (83.48) | 942 (81.44) |  |
| Diabetes |  |  |  | **0.029** |
| Yes | 318 (21.29) | 380 (25.62) | 403 (27.16) |  |
| No | 893 (78.71) | 832 (74.38) | 808 (72.84) |  |
| AST (U/L) | 25.66 (0.86) | 25.35 (0.44) | 25.63 (0.41) | **0.001** |
| ALT (U/L) | 28.86 (0.86) | 29.56 (0.78) | 28.39 (0.57) | 0.168 |
| GGT (U/L) | 35.01 (1.46) | 37.72 (2.11) | 32.68 (0.95) | 0.388 |
| GHB (%) | 5.84 (0.04) | 5.86 (0.04) | 5.86 (0.04) | 0.429 |
| GLU(mmol/L) | 6.27 (0.07) | 6.42 (0.08) | 6.39 (0.08) | **0.007** |
| HDL (mmol/L) | 1.18 (0.01) | 1.24 (0.01) | 1.25 (0.01) | **<0.001** |
| LDL (mmol/L) | 3.09 (0.04) | 3.11 (0.05) | 3.04 (0.04) | 0.530 |
| TC (mmol/L) | 5.06 (0.05) | 5.16 (0.05) | 5.08 (0.05) | 0.437 |
| TG (mmol/L) | 1.79 (0.03) | 1.82 (0.04) | 1.78 (0.05) | 0.406 |
| Platelet (1000 cells/uL) | 255.71 (2.31) | 247.30 (2.82) | 245.66 (2.86) | **0.011** |
| aMED | 5.15 (0.03) | 5.75 (0.03) | 6.42 (0.03) | **<0.001** |
| aMED |  |  |  | **<0.001** |
| T1 | 639 (58.66) | 294 (25.90) | 63 (5.85) |  |
| T2 | 469 (34.79) | 597 (48.54) | 422 (36.24) |  |
| T3 | 103 (6.55) | 321 (25.56) | 726 (57.91) |  |
| HEI-2020 | 40.61 (0.35) | 48.66 (0.31) | 57.89 (0.40) | **<0.001** |
| HEI-2020 |  |  |  | **<0.001** |
| T1 | 799 (68.52) | 334 (30.54) | 78 (7.28) |  |
| T2 | 343 (26.86) | 557 (43.61) | 312 (28.85) |  |
| T3 | 69 (4.63) | 321 (25.85) | 821 (63.87) |  |
| DASH | 24.10 (0.07) | 26.38 (0.07) | 29.09 (0.09) | **<0.001** |
| DASH |  |  |  | **<0.001** |
| T1 | 859 (73.68) | 285 (24.48) | 28 (2.94) |  |
| T2 | 311 (23.98) | 646 (53.72) | 285 (23.89) |  |
| T3 | 41 (2.34) | 281 (21.80) | 898 (73.17) |  |
| DII | 2.13 (0.05) | 1.25 (0.07) | 0.13 (0.07) | **<0.001** |
| DII |  |  |  | **<0.001** |
| T1 | 151 (12.91) | 354 (32.71) | 706 (60.57) |  |
| T2 | 387 (34.62) | 464 (35.65) | 361 (28.87) |  |
| T3 | 673 (52.46) | 394 (31.64) | 144 (10.56) |  |

Continuous variables were expressed as weighted means (SEs), and *p*-values are derived using the Student’s t-test. Categorical variables were expressed as unweighted number (weighted percent), and *p*-values are derived using the chi-square test.
