# Supplementary material for: Association of dietary quality and mortality in the non-alcoholic fatty liver disease and advanced fibrosis populations: NHANES 2005–2018
Source: Front Nutr. 2025 Jan 23;12:1507342. doi: 10.3389/fnut.2025.1507342 (PMC11798782; doi:10.3389/fnut.2025.1507342)
Supplement: Supplementary file 5 [file Table_5.docx]

**Table S5.** Baseline characteristics of NAFLD patients according to the DII score

| Characteristic | T1 | T2 | T3 | *p*-value |
| --- | --- | --- | --- | --- |
| Age (years) | 51.25 (0.58) | 50.14 (0.59) | 49.97 (0.67) | 0.169 |
| Sex |  |  |  | **<0.001** |
| Male | 823(69.49) | 681(56.47) | 517(40.03) |  |
| Female | 388(30.51) | 531(43.53) | 694(59.97) |  |
| Race |  |  |  | **0.002** |
| Non-Hispanic Black | 173(6.30) | 247(10.34) | 280(12.15) |  |
| Non-Hispanic White | 593(73.57) | 579(71.39) | 596(69.82) |  |
| Mexican American | 256(9.26) | 204(9.29) | 150(7.01) |  |
| Other Hispanic | 105(4.94) | 117(4.60) | 124(6.04) |  |
| Other race | 84(5.93) | 65(4.37) | 61(4.99) |  |
| BMI (kg/m^2^) | 34.04 (0.23) | 34.81 (0.24) | 35.04 (0.24) | **<0.001** |
| BMI |  |  |  | **<0.001** |
| <25 (kg/m^2^) | 13(1.00) | 8(0.41) | 15(1.33) |  |
| 25 to < 30 (kg/m^2^) | 311(26.20) | 265(21.54) | 223(17.42) |  |
| ≥30 (kg/m^2^) | 887(72.80) | 939(78.05) | 973(81.26) |  |
| Waist circumference (cm) | 113.54 (0.56) | 114.27 (0.54) | 114.25 (0.52) | 0.184 |
| Hypertension |  |  |  | 0.758 |
| Yes | 243(17.36) | 239(17.12) | 245(15.92) |  |
| No | 968(82.64) | 973(82.88) | 966(84.08) |  |
| Diabetes |  |  |  | 0.483 |
| Yes | 342(23.25) | 374(25.27) | 385(26.07) |  |
| No | 869(76.75) | 838(74.73) | 826(73.93) |  |
| AST (U/L) | 25.63 (0.29) | 26.45 (0.74) | 24.49 (0.68) | **<0.001** |
| ALT (U/L) | 29.49 (0.47) | 30.67 (0.97) | 26.44 (0.67) | **<0.001** |
| GGT (U/L) | 33.86 (0.94) | 36.98 (1.92) | 34.59 (1.82) | **0.012** |
| GHB (%) | 5.82 (0.05) | 5.86 (0.04) | 5.88 (0.04) | 0.523 |
| GLU(mmol/L) | 6.35 (0.09) | 6.40 (0.08) | 6.33 (0.08) | 0.604 |
| HDL (mmol/L) | 1.20 (0.01) | 1.23 (0.01) | 1.25 (0.01) | **0.013** |
| LDL (mmol/L) | 3.03 (0.04) | 3.08 (0.04) | 3.13 (0.05) | 0.364 |
| TC (mmol/L) | 5.05 (0.05) | 5.10 (0.04) | 5.15 (0.05) | 0.326 |
| TG (mmol/L) | 1.85 (0.04) | 1.77 (0.03) | 1.77 (0.04) | 0.198 |
| Platelet (1000 cells/uL) | 243.13 (2.95) | 246.70 (2.38) | 259.56 (2.84) | **<0.001** |
| aMED | 6.22 (0.03) | 5.70 (0.03) | 5.39 (0.03) | **<0.001** |
| aMED |  |  |  | **<0.001** |
| T1 | 146(13.62) | 330(30.61) | 520(46.16) |  |
| T2 | 437(37.37) | 537(42.70) | 514(40.02) |  |
| T3 | 628(49.01) | 345(26.69) | 177(13.82) |  |
| HEI-2020 | 55.61 (0.41) | 48.03 (0.42) | 43.34 (0.38) | **<0.001** |
| HEI-2020 |  |  |  | **<0.001** |
| T1 | 157(13.80) | 383(36.23) | 671(56.68) |  |
| T2 | 375(32.19) | 477(38.26) | 360(29.03) |  |
| T3 | 679(54.01) | 352(25.51) | 180(14.29) |  |
| DASH | 28.22 (0.11) | 26.24 (0.10) | 25.09 (0.09) | **<0.001** |
| DASH |  |  |  | **<0.001** |
| T1 | 155(12.79) | 386(34.85) | 631(53.11) |  |
| T2 | 349(30.12) | 469(37.23) | 424(35.13) |  |
| T3 | 707(57.10) | 357(27.93) | 156(11.75) |  |
| AHEI | 44.57 (0.40) | 37.00 (0.41) | 31.82 (0.29) | **<0.001** |
| AHEI |  |  |  | **<0.001** |
| T1 | 151(11.27) | 387(33.09) | 673(53.51) |  |
| T2 | 354(30.64) | 464(36.57) | 394(34.64) |  |
| T3 | 706(58.10) | 361(30.34) | 144(11.84) |  |

Continuous variables were expressed as weighted means (SEs), and *p*-values are derived using the Student’s t-test. Categorical variables were expressed as unweighted number (weighted percent), and *p*-values are derived using the chi-square test.
