# Supplementary material for: Association of dietary quality and mortality in the non-alcoholic fatty liver disease and advanced fibrosis populations: NHANES 2005–2018
Source: Front Nutr. 2025 Jan 23;12:1507342. doi: 10.3389/fnut.2025.1507342 (PMC11798782; doi:10.3389/fnut.2025.1507342)
Supplement: Supplementary file 6 [file Table_6.docx]

**Table S6** Subgroup analysis of the association of five dietary quality indexes with cardiovascular mortality in patient with NAFLD

| Characteristic | OR | 95%CI | *p*-value | *p* for interaction |
| --- | --- | --- | --- | --- |
| aMED |  |  |  |  |
| Age |  |  |  | 0.453 |
| <50 (years) | 1.474 | 0.164,3.202 | 0.729 |  |
| ≥50 (years) | 0.795 | 0.616,1.025 | 0.076 |  |
| Sex |  |  |  | 0.315 |
| Male | 0.869 | 0.664,1.137 | 0.304 |  |
| Female | 0.688 | 0.440,1.073 | 0.099 |  |
| Race |  |  |  | 0.720 |
| Mexican American | 1.177 | 0.249,5.562 | 0.837 |  |
| Non-HI BLACK | 1.011 | 0.578,1.769 | 0.968 |  |
| Non-HI White | 0.791 | 0.582,1.075 | 0.134 |  |
| Other Hispanic | 0.607 | 0.221,1.671 | 0.334 |  |
| Other races | 0.245 | 0.025,2.393 | 0.226 |  |
| BMI |  |  |  | 0.222 |
| <25(kg/m^2^)^a^ | $-$ | $-$ | $-$ |  |
| 25 to <30 (kg/m^2^) | 1.507 | 0.841,2.699 | 0.168 |  |
| ≥30(kg/m^2^) | 0.717 | 0.544,0.945 | 0.018 |  |
| Hypertension |  |  |  | 0.107 |
| Yes | 1.044 | 0.598,1.821 | 0.880 |  |
| No | 0.727 | 0.540,0.980 | 0.036 |  |
| Diabetes |  |  |  | 0.247 |
| Yes | 0.678 | 0.492,0.935 | 0.018 |  |
| No | 0.918 | 0.553,1.524 | 0.741 |  |
| HEI-2020 |  |  |  |  |
| Age |  |  |  | 0.653 |
| <50 (years) | 0.905 | 0.629,1.302 | 0.591 |  |
| ≥50 (years) | 0.979 | 0.957,1.001 | 0.064 |  |
| Sex |  |  |  | 0.672 |
| Male | 0.982 | 0.962,1.002 | 0.082 |  |
| Female | 0.976 | 0.924,1.032 | 0.392 |  |
| Race |  |  |  | 0.552 |
| Mexican American | 0.931 | 0.843,1.029 | 0.160 |  |
| Non-HI BLACK | 0.992 | 0.936,1.053 | 0.801 |  |
| Non-HI White | 0.983 | 0.957,1.010 | 0.224 |  |
| Other Hispanic | 0.900 | 0.788,1.029 | 0.123 |  |
| Other races | 0.921 | 0.810,1.047 | 0.208 |  |
| BMI |  |  |  | 0.650 |
| <25 (kg/m^2^) | $-$ | $-$ | $-$ |  |
| 25 to <30 (kg/m2) | 1.009 | 0.955,1.066 | 0.755 |  |
| ≥30 (kg/m^2^) | 0.971 | 0.950,0.992 | 0.008 |  |
| Hypertension |  |  |  | 0.086 |
| Yes | 1.003 | 0.963,1.045 | 0.876 |  |
| No | 0.967 | 0.939,0.997 | 0.030 |  |
| Diabetes |  |  |  | 0.578 |
| Yes | 0.971 | 0.945,0.998 | 0.034 |  |
| No | 0.986 | 0.940,1.034 | 0.565 |  |
| DASH |  |  |  |  |
| Age |  |  |  | 0.495 |
| <50 (years) | 1.027 | 0.521,2.024 | 0.939 |  |
| ≥50 (years) | 0.920 | 0.848,0.998 | 0.043 |  |
| Sex |  |  |  | 0.160 |
| Male | 0.959 | 0.884,1.040 | 0.306 |  |
| Female | 0.868 | 0.746,1.011 | 0.069 |  |
| Race |  |  |  | 0.984 |
| Mexican American | 0.752 | 0.426,1.326 | 0.325 |  |
| Non-HI BLACK | 0.915 | 0.701,1.195 | 0.516 |  |
| Non-HI White | 0.943 | 0.854,1.041 | 0.242 |  |
| Other Hispanic | 0.617 | 0.334,1.140 | 0.123 |  |
| Other races | 0.735 | 0.390,1.384 | 0.340 |  |
| BMI |  |  |  | 0.215 |
| <25 (kg/m^2^) | $-$ | $-$ | $-$ |  |
| 25 to <30 (kg/m2) | 1.247 | 0.923,1.684 | 0.150 |  |
| ≥30 (kg/m^2^) | 0.878 | 0.813,0.948 | <0.001 |  |
| Hypertension |  |  |  | 0.226 |
| Yes | 0.965 | 0.862,1.080 | 0.534 |  |
| No | 0.894 | 0.803,0.996 | 0.042 |  |
| Diabetes |  |  |  | 0.777 |
| Yes | 0.916 | 0.828,1.014 | 0.092 |  |
| No | 0.932 | 0.774,1.122 | 0.456 |  |
| AHEI |  |  |  |  |
| Age |  |  |  | 0.236 |
| <50 (years) | 0.952 | 0.751,1.206 | 0.684 |  |
| ≥50 (years) | 0.970 | 0.944,0.996 | 0.026 |  |
| Sex |  |  |  | 0.136 |
| Male | 0.984 | 0.959,1.010 | 0.217 |  |
| Female | 0.948 | 0.896,1.002 | 0.059 |  |
| Race |  |  |  | 0.718 |
| Mexican American | 0.923 | 0.851,1.001 | 0.052 |  |
| Non-HI BLACK | 0.997 | 0.938,1.059 | 0.918 |  |
| Non-HI White | 0.970 | 0.940,1.002 | 0.068 |  |
| Other Hispanic | 0.907 | 0.745,1.103 | 0.327 |  |
| Other races | 0.929 | 0.764,1.130 | 0.464 |  |
| BMI |  |  |  | 0.789 |
| <25 (kg/m^2^) | $-$ | $-$ | $-$ |  |
| 25 to <30 (kg/m2) | 1.007 | 0.950,1.067 | 0.821 |  |
| ≥30 (kg/m^2^) | 0.966 | 0.940,0.992 | 0.011 |  |
| Hypertension |  |  |  | 0.460 |
| Yes | 0.968 | 0.929,1.009 | 0.123 |  |
| No | 0.965 | 0.931,0.999 | 0.046 |  |
| Diabetes |  |  |  | 0.909 |
| Yes | 0.973 | 0.947,1.000 | 0.048 |  |
| No | 0.966 | 0.907,1.028 | 0.274 |  |
| DII |  |  |  |  |
| Age |  |  |  | 0.358 |
| <50 (years) | 3.777 | 0.075,189.692 | 0.506 |  |
| ≥50 (years) | 1.313 | 1.121,1.538 | <0.001 |  |
| Sex |  |  |  | 0.610 |
| Male | 1.262 | 1.062,1.500 | 0.008 |  |
| Female | 1.363 | 1.07,1.735 | 0.012 |  |
| Race |  |  |  | 0.553 |
| Mexican American | 1.484 | 0.656,3.357 | 0.343 |  |
| Non-HI BLACK | 1.059 | 0.670,1.675 | 0.805 |  |
| Non-HI White | 1.351 | 1.120,1.629 | 0.002 |  |
| Other Hispanic | 0.904 | 0.534,1.529 | 0.706 |  |
| Other races | 1.853 | 0.520,6.603 | 0.341 |  |
| BMI |  |  |  | 0.810 |
| <25 (kg/m^2^) | $-$ | $-$ | $-$ |  |
| 25 to <30 (kg/m2) | 1.214 | 0.807,1.827 | 0.352 |  |
| ≥30 (kg/m^2^) | 1.364 | 1.171,1.588 | <0.001 |  |
| Hypertension |  |  |  | 0.310 |
| Yes | 1.176 | 0.946,1.463 | 0.144 |  |
| No | 1.360 | 1.119,1.652 | 0.002 |  |
| Diabetes |  |  |  | 0.673 |
| Yes | 1.285 | 1.066,1.548 | 0.009 |  |
| No | 1.377 | 1.052,1.802 | 0.020 |  |

Adjusted for age, sex, race, BMI, hypertension and diabetes.

^a^ Because there were no NAFLD patients with BMI less than 25 kg/m2 who died from cardiovascular diseases, this row is empty.
